# Maculopapular-Type Drug Eruption Caused by Cetuximab

**DOI:** 10.7759/cureus.102768

**Published:** 2026-02-01

**Authors:** Noriko Ikegawa, Natsuko Saito-Sasaki, Yu Sawada

**Affiliations:** 1 Dermatology, University of Occupational and Environmental Health, Kitakyushu, JPN

**Keywords:** case report, cetsuximab, delayed hypersensitivity, drug eruption, type iv allergy

## Abstract

Cetuximab, a chimeric immunoglobulin G1 (IgG1) monoclonal antibody targeting the epidermal growth factor receptor, is well known to cause immediate hypersensitivity reactions mediated by pre-existing IgE antibodies against galactose-α-1,3-galactose (α-gal). In contrast, T-cell-mediated type IV hypersensitivity reactions are exceedingly rare. We report an 85-year-old woman with stage IV colon cancer who developed a reproducible exanthematous (maculopapular) drug eruption after the re-administration of cetuximab. The eruption occurred several days after infusion, resolved promptly after drug discontinuation, and recurred upon unintentional re-exposure during routine oncologic re-administration. Histopathology revealed interface dermatitis with eosinophilic infiltration, and laboratory findings showed mild eosinophilia. The clinical course and histologic features, including a reproducible eruption upon re-exposure, were strongly consistent with a drug-induced type IV hypersensitivity reaction, supporting the diagnosis. This case underscores the importance of recognizing delayed cutaneous adverse reactions when considering treatment interruption and re-challenge during prolonged or intermittent cetuximab therapy.

## Introduction

Cetuximab is a chimeric (mouse-human) immunoglobulin G1 (IgG1) monoclonal antibody targeting the epidermal growth factor receptor (EGFR) and is indicated for RAS wild-type unresectable or recurrent colorectal and head-and-neck cancers [1,2]. Immediate hypersensitivity reactions to cetuximab, mediated by pre-existing IgE antibodies against galactose-α-1,3-galactose (α-gal), have been well-documented [3]. In contrast, type IV (T-cell-mediated) drug eruptions associated with biologic agents, including EGFR inhibitors, are considered uncommon but are being increasingly recognized as the clinical use of biologics expands.

Most cutaneous adverse reactions to EGFR inhibitors are attributed to the pharmacologic effects of EGFR blockade on keratinocytes rather than immune-mediated mechanisms [4]. Accordingly, delayed-type hypersensitivity reactions to cetuximab have been only rarely reported, and their clinical and histopathologic characteristics remain insufficiently characterized. Nevertheless, recognition of such reactions is clinically important, as delayed immune-mediated eruptions may influence decisions regarding treatment interruption, re-challenge, and long-term safety monitoring in patients receiving biologic therapies.

Herein, we report a rare case of cetuximab-induced type IV hypersensitivity reaction confirmed by reproducible eruption upon re-exposure, and discuss its potential immunologic mechanisms and clinical implications.

## Case presentation

An 85-year-old woman with clinical stage IV colon cancer was treated with cetuximab (500 mg/m² every two weeks) in the Department of Gastroenterology at the University of Occupational and Environmental Health Hospital. After approximately six months of therapy, cetuximab was interrupted because of severe paronychia. Following clinical improvement, cetuximab was re-initiated two months later. Approximately one week after the first re-administration, the patient developed diffuse erythematous macules on the trunk and extremities. Cetuximab was discontinued, and oral fexofenadine (60 mg twice daily) was initiated, resulting in gradual resolution of the eruption.

Cetuximab was restarted after a further two-month interruption, and a similar cutaneous eruption developed more rapidly, within two days after infusion. The shortened latency period upon the second re-exposure suggested the involvement of immunologic memory. At the time of both eruptions, no new medications had been introduced, and the patient had been receiving stable concomitant drugs for several months, making other drug-induced eruptions unlikely. Viral exanthems and paraneoplastic skin eruptions were also considered; however, the absence of systemic viral symptoms and the reproducible temporal relationship with cetuximab administration argued against these possibilities.

**Figure 1:**
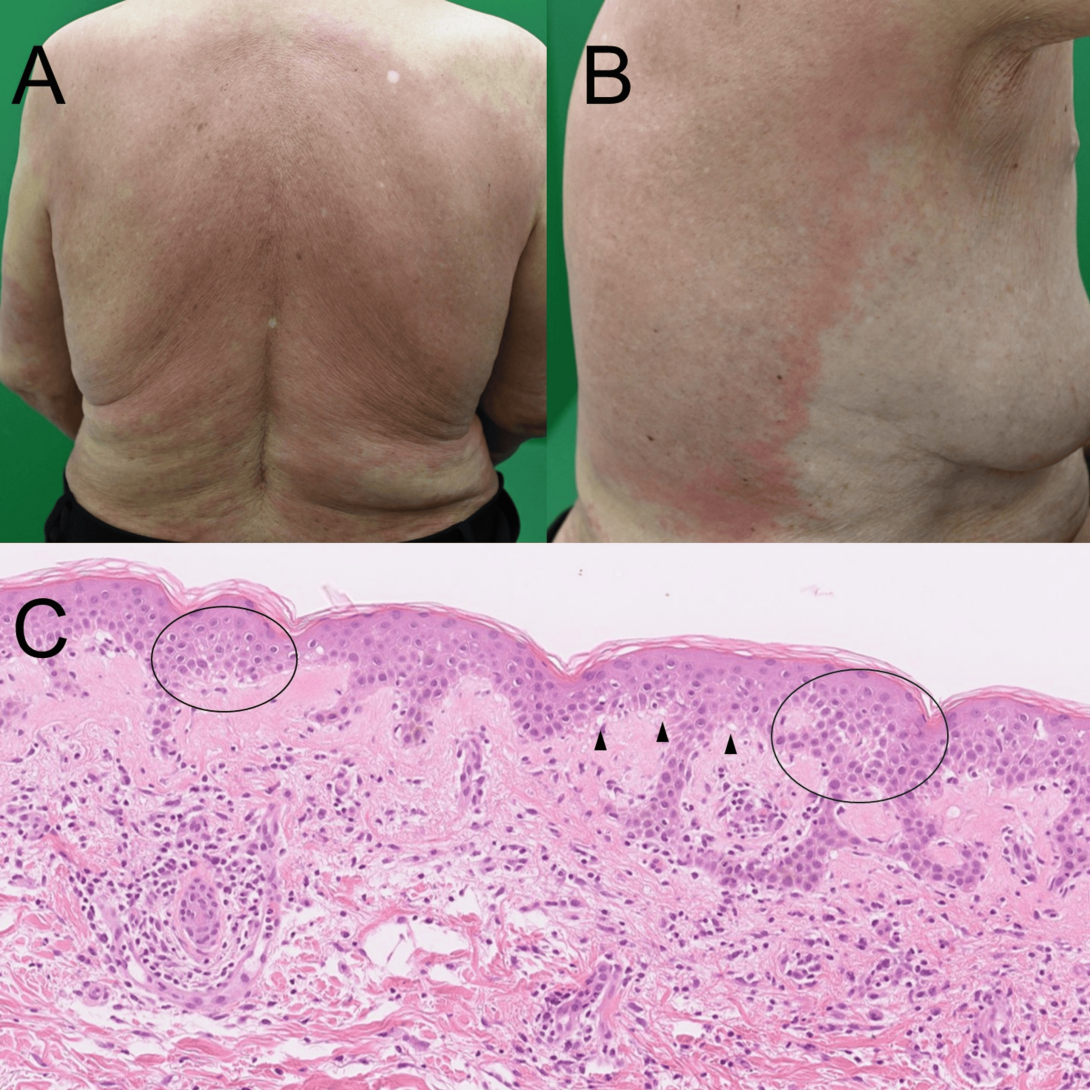
Clinical manifestation and histological examination (A, B) Diffuse erythematous macules and patches on the back and lateral trunk approximately one week after re-administration of cetuximab. The eruption was confluent and symmetric, without pustules or mucosal involvement. (C) Histopathologic examination of an erythematous lesion showing mild epidermal spongiosis (black circles) and basal vacuolar alteration (arrowheads), consistent with a drug-induced eruption (hematoxylin and eosin stain, ×100).

The patient was referred to our dermatology department for further evaluation. Physical examination revealed pruritic erythematous macules and papules on the back, abdomen, and lateral chest without mucosal involvement (Figures 1A, 1B). Laboratory testing showed a normal white blood cell count (7,100/μL) with mild eosinophilia (8.8%; absolute eosinophil count, approximately 625/μL). Other hematologic parameters were within normal limits. Serum biochemistry revealed no hepatic or renal dysfunction, although a mild elevation of C-reactive protein (2.04 mg/dL) was noted.

A skin biopsy obtained from an erythematous lesion on the trunk demonstrated mild epidermal spongiosis, basal vacuolar alteration, and superficial perivascular lymphocytic infiltration with scattered eosinophils (Figure 1C). The reproducible eruption upon re-exposure, together with the histopathologic findings and progressively shortened latency, was strongly consistent with a cetuximab-induced type IV hypersensitivity reaction.

After the second eruption, cetuximab was permanently discontinued. The skin eruption resolved with continued oral fexofenadine alone, without systemic corticosteroids, and eosinophil counts normalized within three weeks after drug withdrawal. No recurrence of cutaneous eruption or eosinophilia was observed during subsequent follow-up under alternative cancer therapy.

## Discussion

Most cutaneous adverse reactions to EGFR inhibitors are attributed to the pharmacologic effects of an EGFR blockade on keratinocyte proliferation and differentiation rather than to immune-mediated mechanisms [4]. These reactions typically present as acneiform eruptions characterized by follicular papules and pustules, reflecting altered epidermal homeostasis. In contrast, the present case demonstrated an exanthematous eruption with interface dermatitis and eosinophilic infiltration, findings that are inconsistent with classic EGFR inhibitor-related acneiform eruptions and instead support an immune-mediated process. Although a previous report described a cetuximab-associated necrotic eruption with neutrophil-dominant inflammation and epidermal necrosis [5], delayed-type hypersensitivity reactions to EGFR inhibitors remain distinctly uncommon.

An emerging concept in the field of biologic therapies is the loss of immune tolerance following treatment interruption, which may predispose patients to immune-mediated adverse events upon re-exposure. Continuous antigen exposure has been proposed to promote peripheral tolerance, whereas prolonged drug-free intervals may permit the recovery or expansion of drug-reactive T-cell clones [6]. This framework is particularly relevant for chimeric monoclonal antibodies, such as cetuximab, and provides an important context for interpreting the clinical course observed in the present case.

Immediate hypersensitivity reactions to cetuximab have been extensively investigated and are well-established to result from pre-existing immunoglobulin E antibodies against galactose-α-1,3-galactose (α-gal), leading to first-infusion anaphylaxis [3]. By contrast, T-cell-mediated type IV hypersensitivity reactions to cetuximab and other EGFR inhibitors are rarely reported. This apparent rarity is notable because cetuximab contains murine-derived components, which might theoretically increase immunogenicity and favor T-cell-driven responses. However, the immunologic mechanisms discussed below should be regarded as proposed hypotheses rather than evidence of causality in this individual case.

Large, highly folded glycoproteins, such as monoclonal antibodies, are inefficiently processed for major histocompatibility complex (MHC) class II presentation, as endolysosomal unfolding and proteolytic cleavage represent critical rate-limiting steps in antigen processing [7]. Consequently, these molecules predominantly expose conformational rather than linear peptide epitopes and may escape the proteolytic degradation required for effective CD4⁺ T-cell priming [8,9]. These characteristics may help explain, at a theoretical level, why delayed-type hypersensitivity reactions to cetuximab are infrequently observed, but they do not establish a direct mechanistic link in the present case.

α-gal sensitization further shapes the immune response to cetuximab [10,11]. In sensitized individuals, the α-gal epitope on the Fab region of cetuximab can rapidly activate mast cells via IgE cross-linking, producing an immediate, T helper 2 (Th2)-skewed effector response [12]. While localized IgE-dominant responses may transiently bias immune reactivity toward immediate hypersensitivity pathways and potentially limit concurrent T helper 1 (Th1)-type cellular responses [13], this remains a speculative explanation and should not be interpreted as causal.

In the present patient, the concept of loss of tolerance provides a unifying explanation for the clinical findings. Repeated interruption and re-administration of cetuximab may have facilitated reactivation of drug-specific memory T-cells, leading to a progressively shortened latency upon re-exposure that is consistent with immunologic memory rather than proof of a specific pathogenic mechanism. This temporal pattern distinguishes the present case from typical pharmacologic EGFR inhibitor-related eruptions.

Several alternative diagnoses were considered. Viral exanthems were unlikely because of the absence of systemic symptoms and the reproducible temporal relationship with cetuximab administration. Paraneoplastic eruptions were also considered; however, the eruption closely correlated with drug exposure rather than tumor progression and resolved promptly after drug withdrawal. Pharmacologic EGFR inhibitor-related acneiform eruptions were considered less likely, given the non-follicular morphology, absence of pustules, delayed onset after long-term therapy, and histopathologic findings of interface dermatitis with eosinophils.

Importantly, the histopathologic findings observed in this case are supportive but not pathognomonic for drug-induced type IV hypersensitivity. Therefore, the diagnosis was established through clinicopathologic correlation, integrating clinical morphology, reproducible temporal association with cetuximab re-exposure, shortened latency, and resolution after drug discontinuation, rather than histology alone.

Alternative diagnostic approaches for delayed drug hypersensitivity, such as patch testing or lymphocyte transformation testing, were considered. However, these tests have limited sensitivity for biologic agents and were not pursued in this elderly patient with advanced malignancy, particularly given the highly reproducible clinical course and clear response to drug withdrawal.

## Conclusions

Although cetuximab is most commonly associated with immediate, α-gal-mediated hypersensitivity reactions, this case illustrates that T-cell-mediated type IV drug eruptions can also occur. In this patient, alternative diagnoses, including viral exanthems, paraneoplastic eruptions, drug-drug interaction-related eruptions, and EGFR inhibitor-associated inflammatory rashes unrelated to hypersensitivity, were carefully considered but were deemed less likely based on the clinical morphology, reproducible temporal relationship with cetuximab re-exposure, histopathologic findings, and prompt resolution after drug withdrawal. These findings support the diagnosis of cetuximab-induced delayed hypersensitivity. Close interdisciplinary collaboration between dermatologists and oncologists is essential to ensure accurate diagnosis, appropriate management, and safe decision-making regarding treatment interruption or re-challenge.
